# Crucial Role of Pre-antimicrobial Cultures and Histopathological Correlation in the Diagnosis of Culture-Negative Endocarditis: A Case Report

**DOI:** 10.7759/cureus.107179

**Published:** 2026-04-16

**Authors:** Cristian J Sosa-Alvarez, Daniela Cordoba-Alvarado, Oriana Y Mariscal-Diaz, Domingo A Manriquez-Vazquez, Carlos E Jimenez-Lopez

**Affiliations:** 1 Cardiology, Instituto de Seguridad y Servicios Sociales de los Trabajadores del Estado, Zapopan, MEX; 2 Internal Medicine, Secretaría de Salud, Zapopan, MEX

**Keywords:** bicuspid aortic valve disease, blood culture-negative endocarditis, histopathology and microbiology, infective endocarditis, surgical aortic valve replacement (savr)

## Abstract

Infective endocarditis (IE) is a life-threatening infection involving the cardiac valves and endocardium, most commonly associated with streptococci and enterococci in patients with underlying structural cardiac abnormalities. The bicuspid aortic valve (BAV) represents a significant predisposing valvular condition compared with patients with a tricuspid aortic valve. Culture-negative endocarditis accounts for a substantial portion of all cases, often attributed to prior antibiotic exposure, and represents a major diagnostic challenge in which histopathological examination of the surgical specimen becomes the decisive diagnostic tool.

We report the case of an elderly male with a known BAV who presented with recurring fever episodes and progressive acute decompensated heart failure. Transthoracic echocardiography demonstrated a highly mobile vegetation on a calcified aortic valve, with severe aortic regurgitation. Empirical antibiotic therapy was initiated prior to surgical referral. Given clinical deterioration, aortic valve replacement was performed. Surgical valve cultures yielded no bacterial growth. Histopathological examination of the resected specimen revealed coagulative necrosis, dystrophic calcification, and Gram-positive structures, confirming the diagnosis of bacterial IE.

This case underscores the critical importance of obtaining blood cultures before initiating antibiotic therapy. Furthermore, it highlights the role of histopathological examination when microbiological cultures fail and reinforces the need for a multidisciplinary approach in the management of IE on a BAV.

## Introduction

Infective endocarditis (IE) is an infection involving the epithelium of the heart valves, the endocardium, or the intima of the aorta and other large or small blood vessels [1]. IE primarily affects young adults; the most common associated pathogens are streptococci and enterococci, and the condition is generally related to underlying cardiac abnormalities such as congenital heart disease and rheumatic heart disease [2].

The bicuspid aortic valve (BAV) consists of two cusps and two intervalvular triangular structures, with one cusp appearing divided into two parts by a raphe, beneath which the intervalvular triangle is absent [3]. BAV is the most common aortic valvulopathy, with a prevalence of 0.5% to 2%, and is more frequent in men, at a 3:1 ratio. Late complications of BAV include aortic regurgitation or stenosis, IE, and aortic dilation and dissection [4].

In cases where blood cultures yield no growth, histopathological examination of the surgical specimen remains the gold standard for the diagnosis of IE, allowing direct visualization of microorganisms and valvular tissue destruction, even in the absence of microbiological confirmation [5]. Culture-negative endocarditis accounts for up to 30% of all IE cases, most commonly attributed to prior antibiotic exposure before blood culture collection or infection with fastidious or nonculturable microorganisms [6]. In this clinical scenario, the integration of microbiological, imaging, and pathological findings becomes essential for establishing a definitive diagnosis and guiding therapeutic decisions.

Delayed or missed diagnosis in culture-negative IE carries significant clinical consequences, as diagnostic delay has been independently associated with increased mortality, higher rates of embolic complications, and a greater likelihood of irreversible valvular destruction requiring urgent surgical intervention [5,6]. This underscores the critical need for a systematic and multidisciplinary diagnostic approach in all patients with suspected IE, regardless of microbiological culture results.

## Case presentation

A 65-year-old male patient with a known BAV was admitted approximately three months prior to his final hospitalization with recurring fever episodes. During the initial workup, cholecystitis was identified alongside an unspecified cardiac murmur. The patient underwent cholecystectomy without complications and was discharged home. Approximately 10 days following discharge, the patient developed progressive dyspnea, with functional class deterioration to NYHA III, peripheral edema, and intermittent oppressive chest pain of moderate intensity, prompting readmission for further evaluation of the previously identified cardiac murmur.

A transthoracic echocardiogram (TTE) revealed a fibrotic aortic valve with moderate calcification and a highly mobile, rounded mass measuring 12 × 8 mm, compatible with vegetation. These findings were consistent with IE with aortic valve involvement, and empirical therapy with vancomycin was initiated.

**Figure 1:**
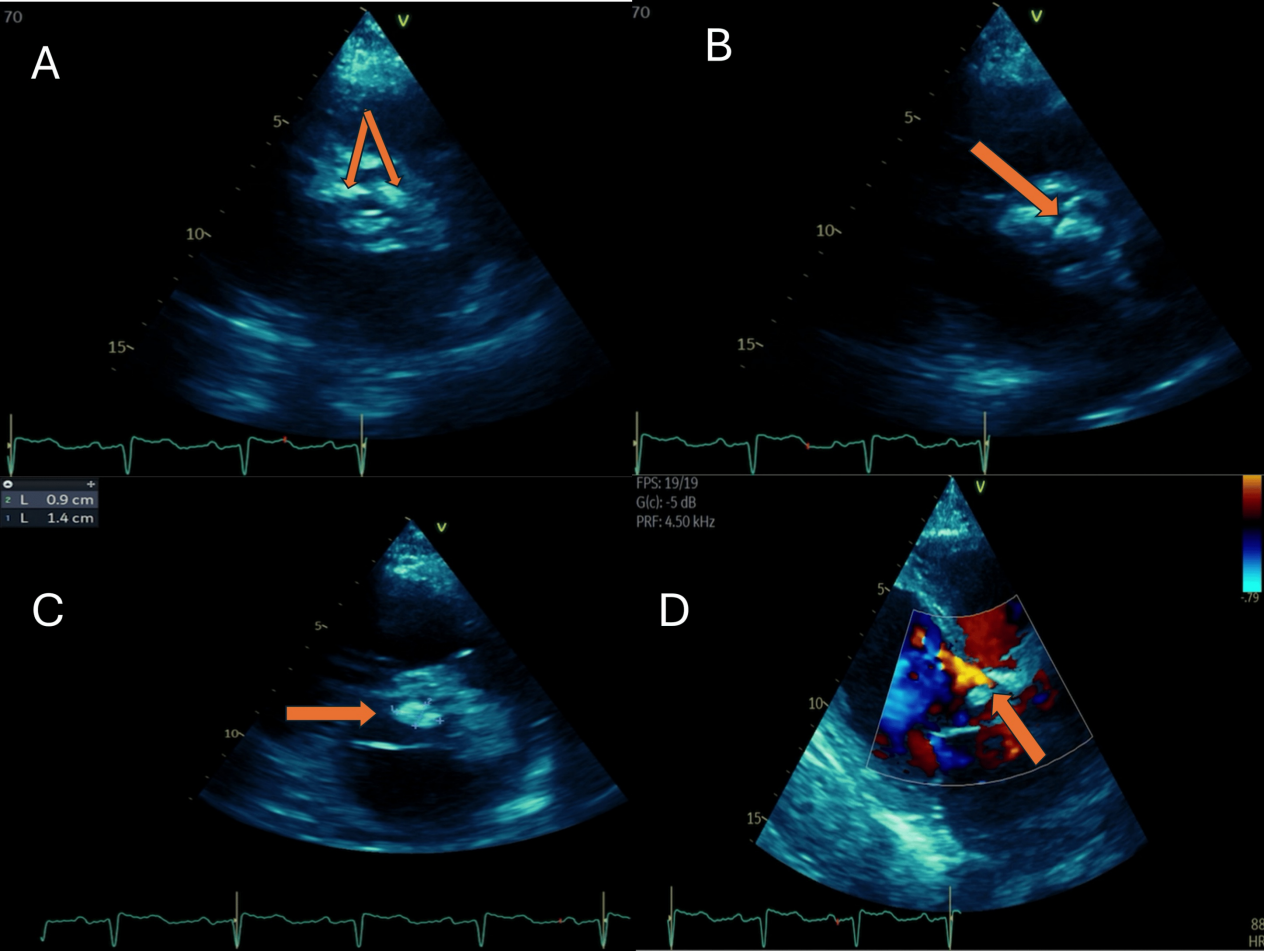
Transthoracic echocardiogram (A) Parasternal short-axis view at the level of the great vessels, demonstrating the aortic valve at the center of the image. A bicuspid aortic valve is identified, with fusion of the right and left commissures, thickened leaflets, and significant calcification. Orange arrows indicate the thickened and calcified valve leaflets. (B) Parasternal long-axis view, demonstrating the aortic valve with a hyperechoic mobile structure suggestive of vegetation. The orange arrow indicates the echogenic mass attached to the aortic valve leaflet, consistent with infectious vegetation. (C) Parasternal long-axis view with measurement of the vegetation, demonstrating dimensions of 0.9 × 1.4 cm. The orange arrow indicates the vegetation on the aortic valve. (D) Color Doppler demonstrating an eccentric aortic regurgitation jet originating at the level of the aortic valve. The turbulent, mosaic color pattern is consistent with severe aortic regurgitation secondary to valvular destruction. The orange arrow indicates the eccentric regurgitant jet extending into the left ventricular outflow tract.

**Figure 2:**
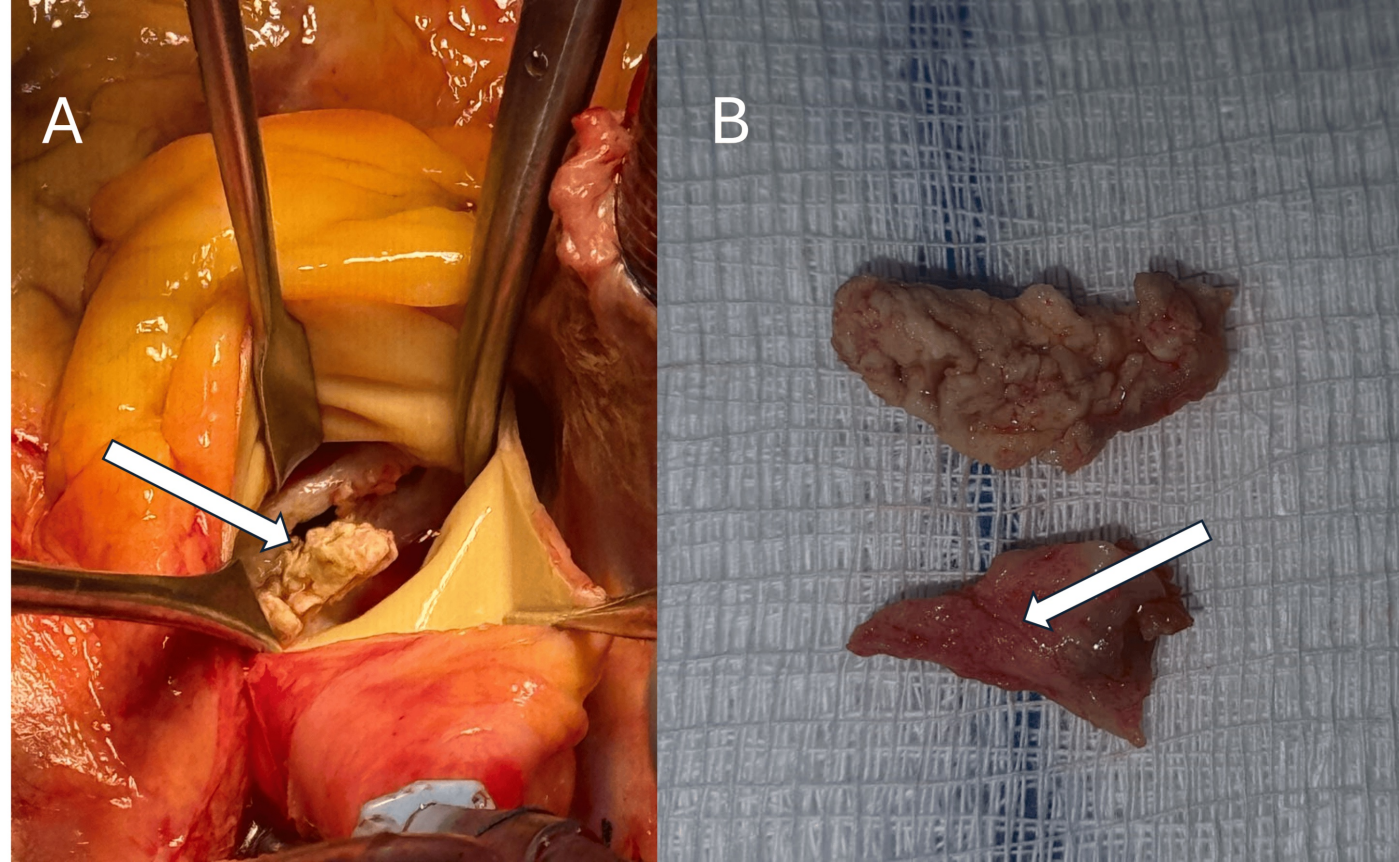
Intraoperative and gross pathological findings (A) Intraoperative photograph demonstrating the surgeon's direct view of the aortic valve, revealing severe valvular destruction with significant calcification and a coaptation defect of the valve leaflets. The white arrow indicates the vegetation measuring 10 × 15 mm attached to the aortic valve. (B) Gross pathological specimen demonstrating the resected aortic valve leaflets. The upper fragment corresponds to the non-coronary cusp, showing extensive calcification, structural destruction, and adherent vegetation. The lower fragment demonstrates the fused left and right coronary cusps, with the characteristic raphe, consistent with bicuspid aortic valve morphology. The white arrow indicates the raphe, confirming the bicuspid morphology of the resected valve.

Given clinical deterioration, the patient was referred to our institution, presenting with acute decompensated heart failure requiring vasopressor support. Three sets of blood cultures were obtained from separate peripheral venipuncture sites, with a minimum interval of 30 to 60 minutes between the first and last draws. A repeat TTE confirmed aortic valve vegetation with severe regurgitation (Figure 1), and a multidisciplinary decision for surgical intervention was made. Intraoperative findings included global cardiomegaly, severe left ventricular dilation, commissural fusion, severe annular sclerosis and calcification, destruction of the non-coronary cusp, and a vegetation on the left coronary cusp measuring 10 × 15 mm (Figure 2). Aortic valve replacement was performed with a St. Jude mechanical prosthesis No. 21.

**Figure 3:**
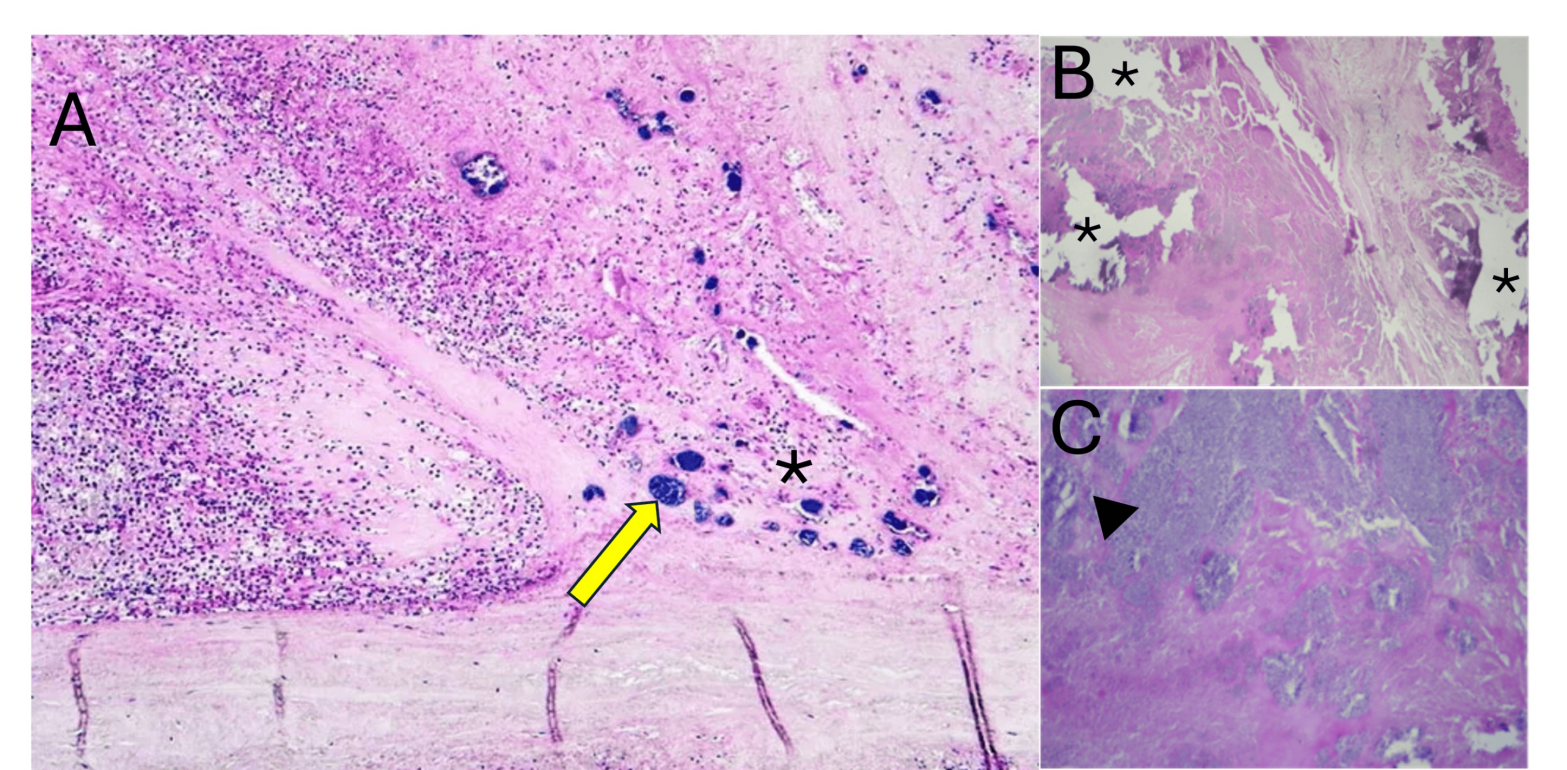
Histopathological findings of the resected aortic valve specimen (A) Hematoxylin and eosin stain demonstrating coagulative eosinophilic necrosis with a dense mixed inflammatory infiltrate composed of lymphocytes and polymorphonuclear leukocytes. The yellow arrow indicates basophilic bacterial colonies with well-defined borders, consistent with Gram-positive organisms. The asterisk (*) denotes areas of coagulative eosinophilic necrosis. (B) Higher magnification demonstrating extensive areas of coagulative necrosis with fibrin deposition. Asterisks (*) indicate multiple foci of dystrophic calcification, irregularly distributed throughout the valvular tissue. (C) The arrowhead (▶) highlights the dense inflammatory infiltrate with areas of tissue destruction, consistent with active infective endocarditis of bacterial etiology.

According to the modified Duke criteria, the present case was classified as possible IE, fulfilling one major criterion - echocardiographic evidence of endocardial involvement with aortic valve vegetation and severe aortic regurgitation - and two minor criteria: a predisposing cardiac condition (BAV) and fever. Following surgical intervention, the resected valve specimen was sent for microbiological culture and histopathological analysis. After seven days of incubation, valve cultures and blood cultures yielded no bacterial growth. Histopathological examination revealed coagulative eosinophilic necrosis, intense basophilic colonies, dystrophic calcification - a characteristic finding of BAV with chronic infectious process - and, on Gram staining, rounded grouped structures compatible with Gram-positive organisms (Figure 3), confirming the diagnosis of bacterial IE despite negative microbiological cultures.

## Discussion

Patients with structural cardiac abnormalities, particularly valvular defects such as BAV, are at higher risk of developing IE [7].

As previously mentioned, the BAV is the most common aortic valvulopathy, with a prevalence of 0.5% to 2%, and is more frequent in men, at a 3:1 ratio [3]. IE in patients with a BAV occurs predominantly in young adults and is associated with severe valvular and perivalvular damage, with aortic abscess reported in up to 50% of cases [8]. The relative risk of developing IE in patients with a BAV is 23.1 times higher compared with patients with a tricuspid aortic valve. Furthermore, a relative risk of 15.6 has been reported in BAV patients under 65 years of age [7], a finding particularly relevant to our patient, a 65-year-old male with a known BAV, who presented with severe valvular destruction of the non-coronary cusp and significant aortic regurgitation secondary to IE, consistent with the aggressive valvular and perivalvular involvement described in the literature for this population.

The risk of developing endocarditis in patients with BAV is primarily attributed to the abnormal shear stress generated by turbulent blood flow across the bicuspid valve, which over time causes endothelial tissue damage, with fibrinogen and platelet deposition leading to fibrosis and calcification, thereby increasing the risk of vegetation formation on the valve leaflets [9,10].

Culture-negative endocarditis accounts for up to 30% of all IE cases, most commonly attributed to prior antibiotic exposure before blood culture collection or infection with fastidious or nonculturable microorganisms [6]. IE in BAV is associated with a high rate of negative blood cultures, particularly in the context of antibiotic administration prior to hospitalization [8]. This was precisely the scenario encountered in our patient, in whom empirical vancomycin therapy had been initiated before surgical intervention, likely accounting for the absence of bacterial growth in both blood and surgical valve cultures.

Histopathological examination of surgical valve specimens remains the gold standard for the diagnosis of IE, particularly when microbiological cultures fail to identify a causative pathogen [5]. In the present case, analysis of the resected aortic valve revealed coagulative necrosis, dystrophic calcification, a mixed inflammatory infiltrate composed of lymphocytes and polymorphonuclear leukocytes, and Gram-positive bacterial colonies with well-defined borders and intense basophilic staining - findings collectively consistent with active IE of bacterial etiology. These histopathological findings proved decisive in establishing a definitive diagnosis in the context of negative blood and valve cultures, confirming the critical and irreplaceable role of surgical pathology in the diagnostic workup of culture-negative IE (Table 1) [5].

**Table 1:** Diagnostic correlation and limitations in a case of culture-negative infective endocarditis

Diagnostic Tool	Result in This Case	Clinical Limitation or Rationale
Blood Cultures	Negative	Prior exposure to vancomycin therapy [8]
Transthoracic Echocardiography	Positive (14 x 9 mm vegetation)	Identification of lesion without microbiological data [5]
Modified Duke Criteria	Possible infective endocarditis (IE) (1 major, 2 minor)	Reduced sensitivity due to negative cultures [6]
Intraoperative Findings	Positive (non-coronary cusp destruction)	Macroscopic confirmation without etiological diagnosis [6]
Histopathological Examination	Definitive (Gram-positive organisms)	Gold standard for diagnosis in culture-negative cases [8]

## Conclusions

This case illustrates the diagnostic complexity of culture-negative IE in a patient with a BAV, where the integration of clinical, echocardiographic, surgical, and histopathological findings proved essential to establish a definitive diagnosis. The BAV, as the most common predisposing structural cardiac condition for IE, should prompt early clinical suspicion and systematic diagnostic evaluation in any patient presenting with fever and cardiac symptoms. When blood cultures yield no growth, histopathological examination of the surgical specimen remains the gold standard and the decisive diagnostic tool capable of identifying microorganisms and confirming active infection, even in the absence of microbiological confirmation.

This case further reinforces the critical importance of obtaining blood cultures before initiating antibiotic therapy, as premature antimicrobial exposure remains the leading cause of culture-negative endocarditis and may significantly delay definitive diagnosis. A multidisciplinary approach integrating cardiology, cardiac surgery, infectious diseases, and pathology is paramount in the management of these challenging cases.
